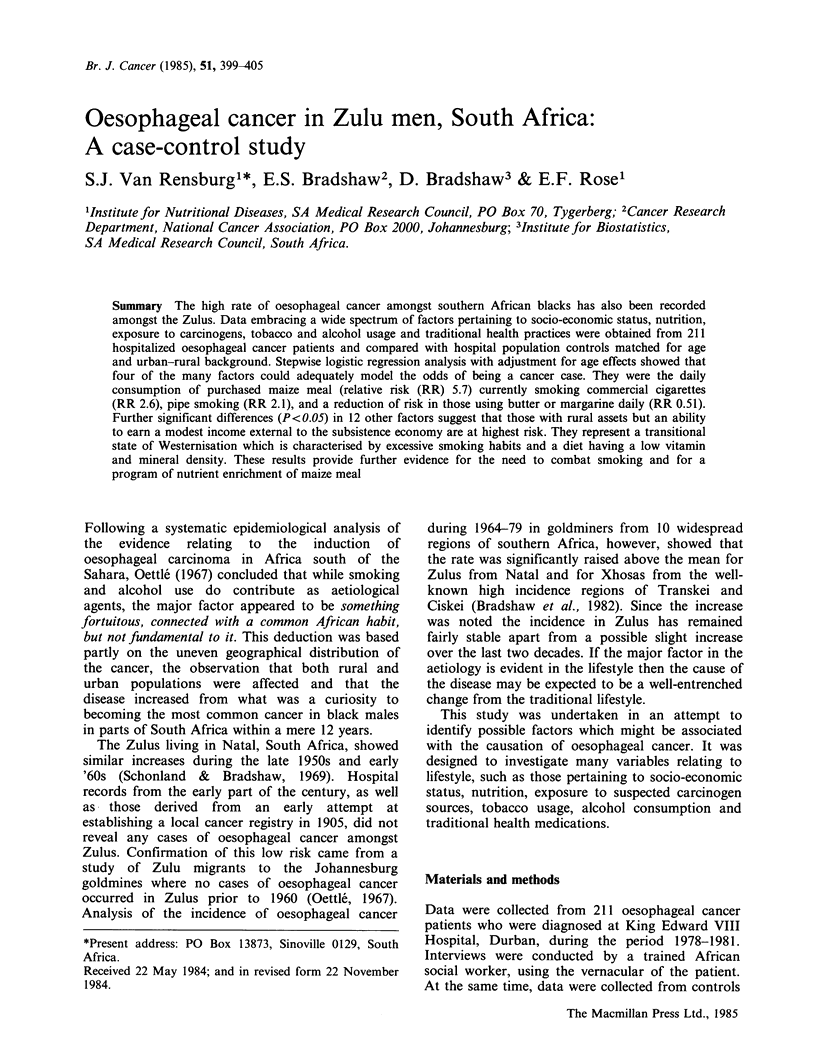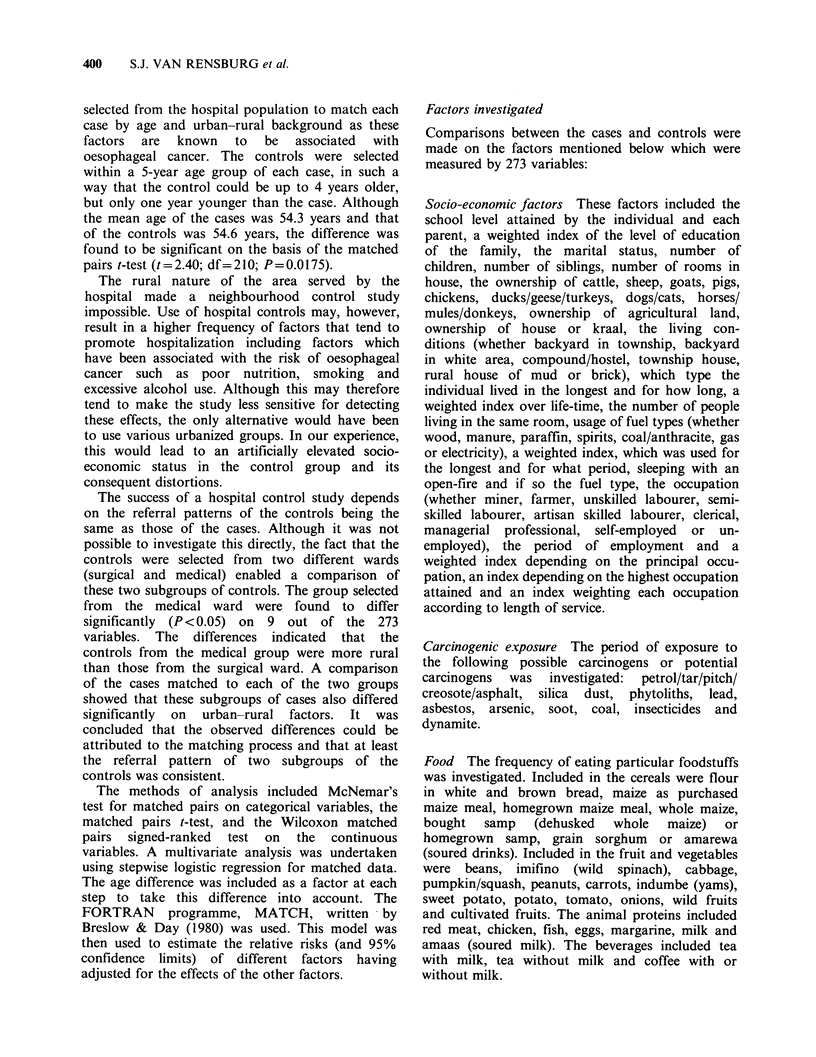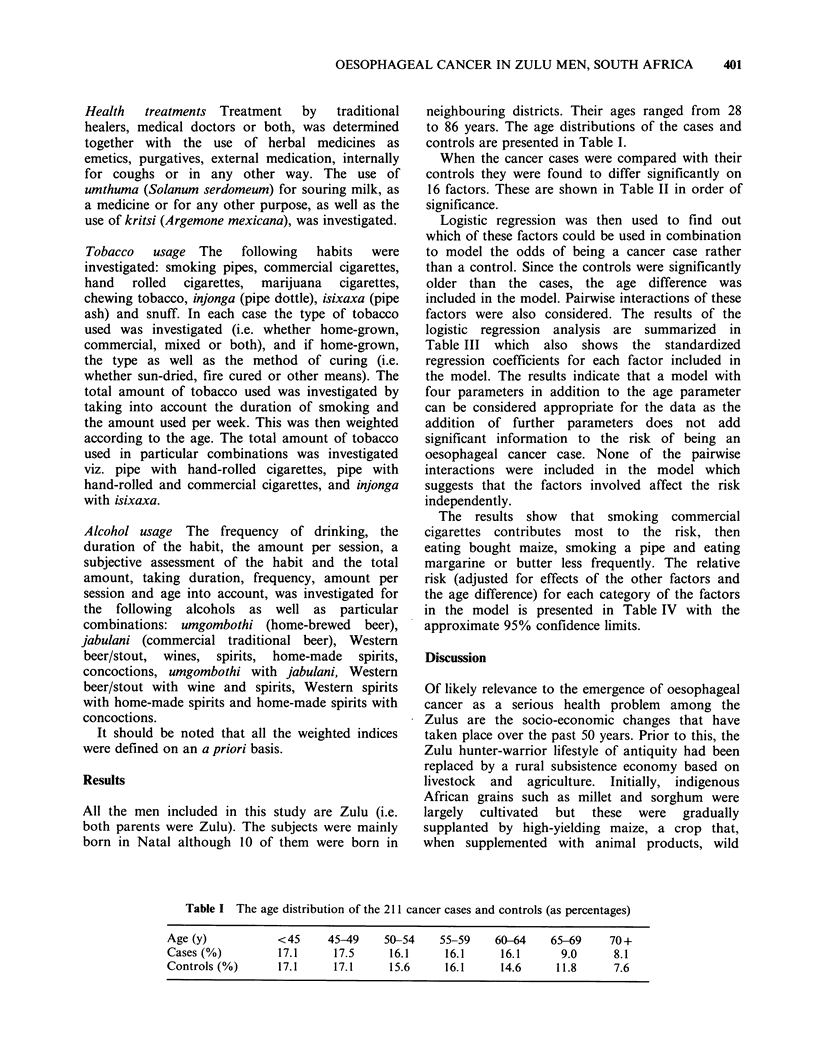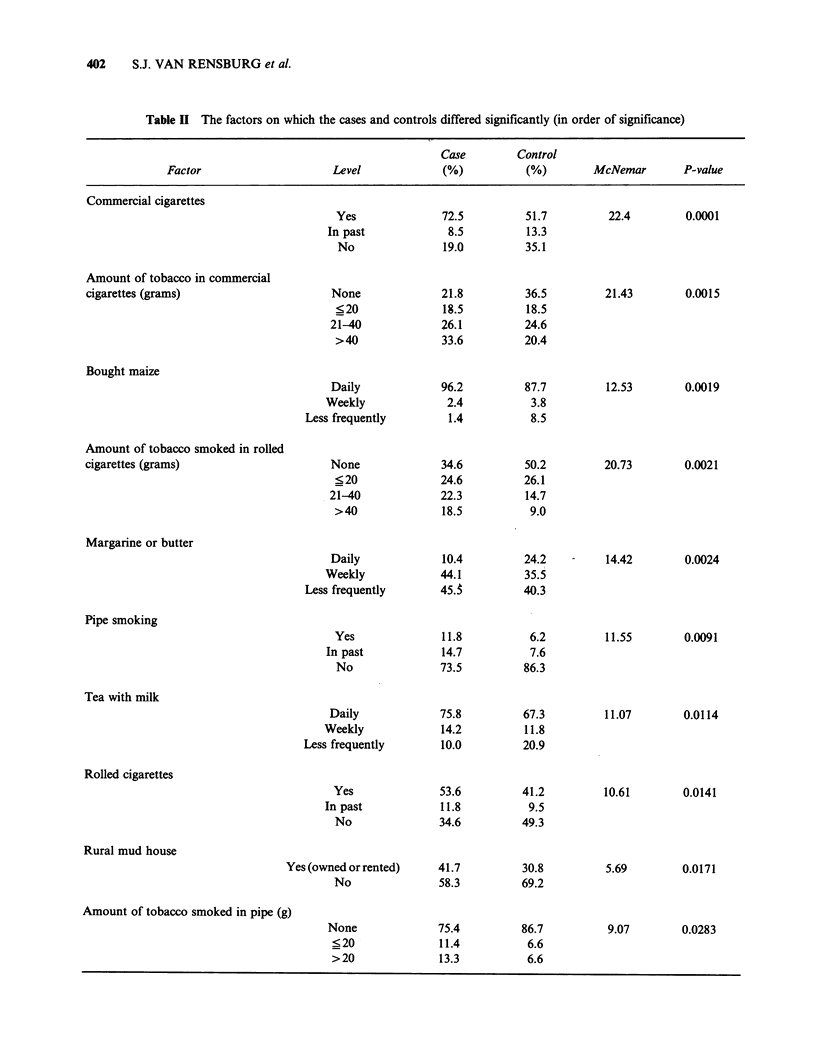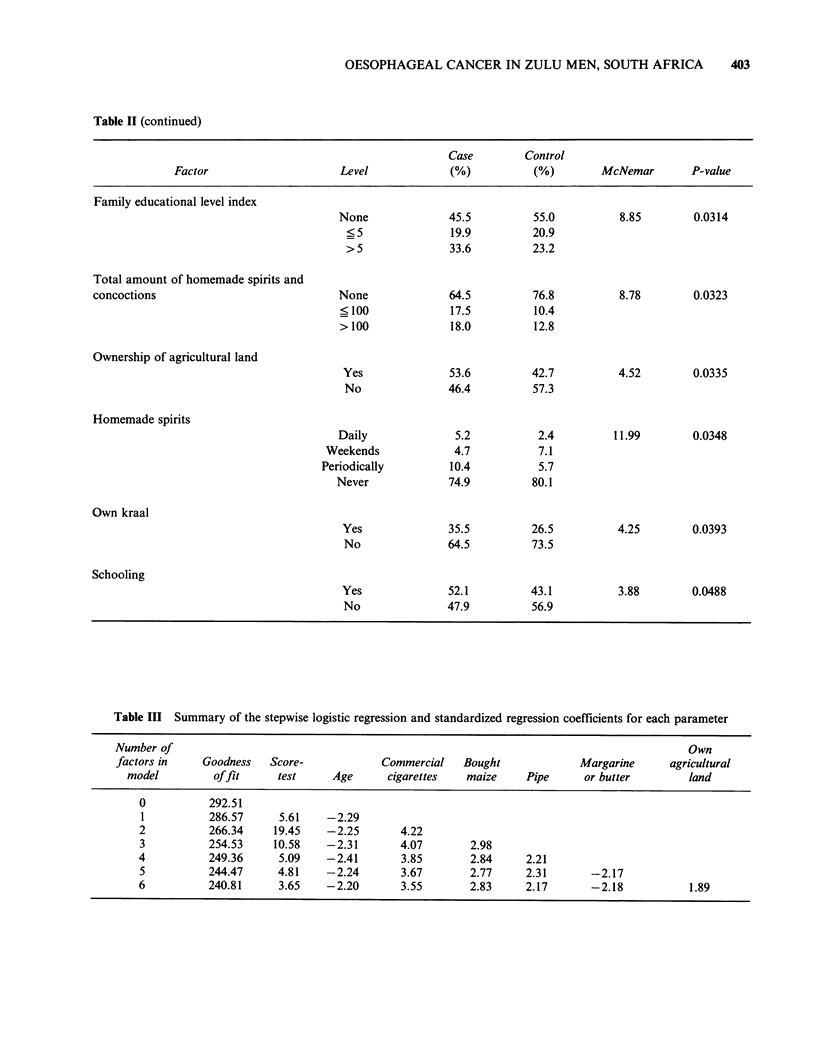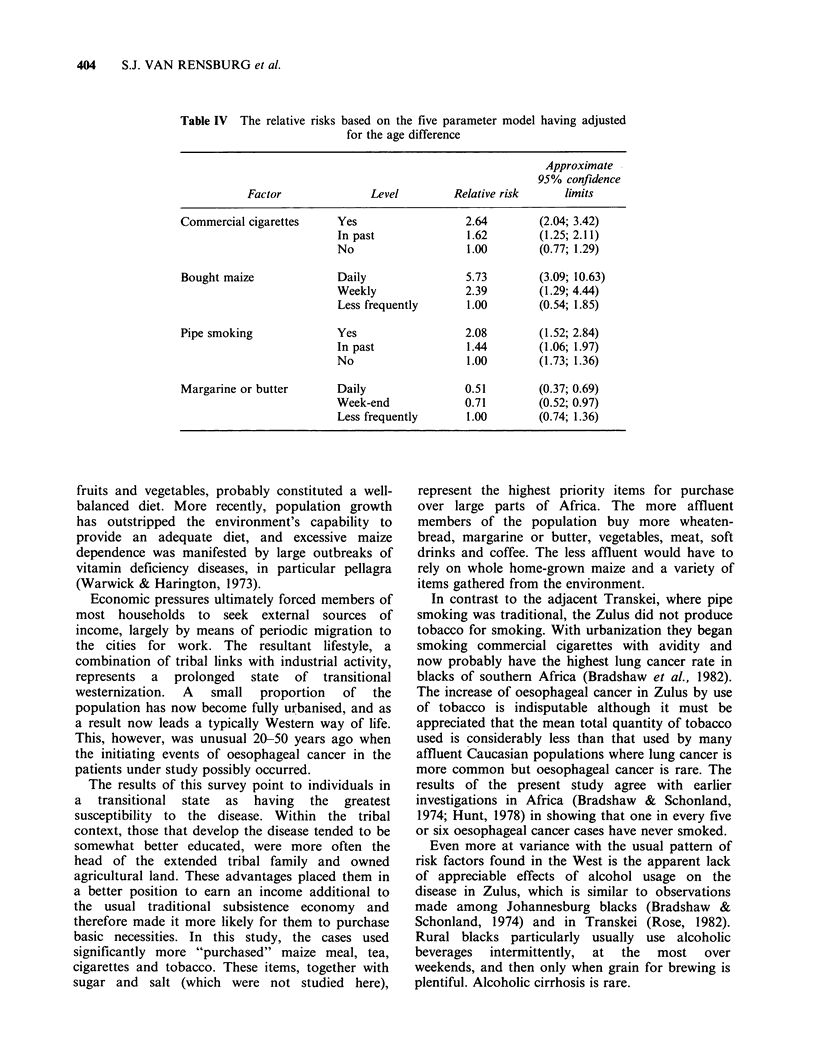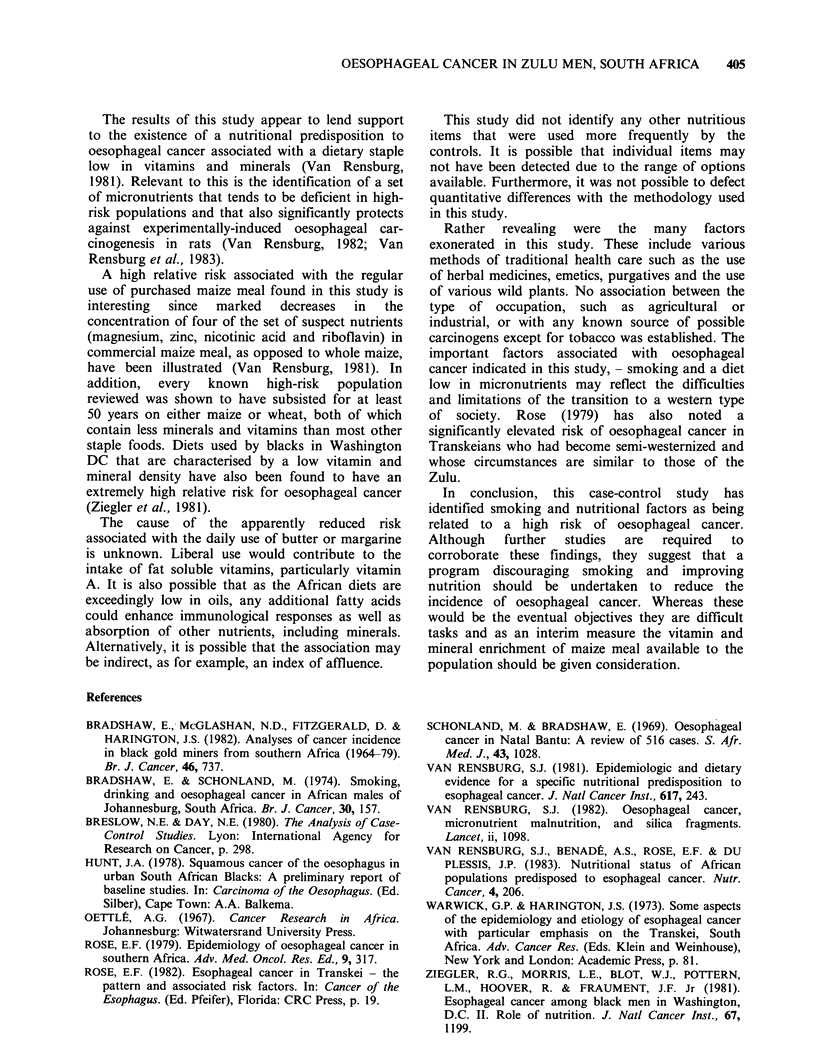# Oesophageal cancer in Zulu men, South Africa: a case-control study.

**DOI:** 10.1038/bjc.1985.54

**Published:** 1985-03

**Authors:** S. J. Van Rensburg, E. S. Bradshaw, D. Bradshaw, E. F. Rose

## Abstract

The high rate of oesophageal cancer amongst southern African blacks has also been recorded amongst the Zulus. Data embracing a wide spectrum of factors pertaining to socio-economic status, nutrition, exposure to carcinogens, tobacco and alcohol usage and traditional health practices were obtained from 211 hospitalized oesophageal cancer patients and compared with hospital population controls matched for age and urban-rural background. Stepwise logistic regression analysis with adjustment for age effects showed that four of the many factors could adequately model the odds of being a cancer case. They were the daily consumption of purchased maize meal (relative risk (RR) 5.7) currently smoking commercial cigarettes (RR 2.6), pipe smoking (RR 2.1), and a reduction of risk in those using butter or margarine daily (RR 0.51). Further significant differences (P less than 0.05) in 12 other factors suggest that those with rural assets but an ability to earn a modest income external to the subsistence economy are at highest risk. They represent a transitional state of Westernisation which is characterised by excessive smoking habits and a diet having a low vitamin and mineral density. These results provide further evidence for the need to combat smoking and for a program of nutrient enrichment of maize meal.


Br. J. Cancer (1985), 51, 399–405

### Oesophageal cancer in Zulu men, South Africa: A case-control study

S.J. Van Rensburg[1]*, E.S. Bradshaw[2], D. Bradshaw[3] & E.F. Rose[1]

[1]*Institute for Nutritional Diseases, SA Medical Research Council, PO Box 70, Tygerberg;* [2]*Cancer Research Department, National Cancer Association, PO Box 2000, Johannesburg;* [3]*Institute for Biostatistics, SA Medical Research Council, South Africa.*

**Summary** The high rate of oesophageal cancer amongst southern African blacks has also been recorded amongst the Zulus. Data embracing a wide spectrum of factors pertaining to socio-economic status, nutrition, exposure to carcinogens, tobacco and alcohol usage and traditional health practices were obtained from 211 hospitalized oesophageal cancer patients and compared with hospital population controls matched for age and urban–rural background. Stepwise logistic regression analysis with adjustment for age effects showed that four of the many factors could adequately model the odds of being a cancer case. They were the daily consumption of purchased maize meal (relative risk (RR) 5.7) currently smoking commercial cigarettes (RR 2.6), pipe smoking (RR 2.1), and a reduction of risk in those using butter or margarine daily (RR 0.51). Further significant differences ($P<0.05$) in 12 other factors suggest that those with rural assets but an ability to earn a modest income external to the subsistence economy are at highest risk. They represent a transitional state of Westernisation which is characterised by excessive smoking habits and a diet having a low vitamin and mineral density. These results provide further evidence for the need to combat smoking and for a program of nutrient enrichment of maize meal

Following a systematic epidemiological analysis of the evidence relating to the induction of oesophageal carcinoma in Africa south of the Sahara, Oettlé (1967) concluded that while smoking and alcohol use do contribute as aetiological agents, the major factor appeared to be *something fortuitous, connected with a common African habit, but not fundamental to it.* This deduction was based partly on the uneven geographical distribution of the cancer, the observation that both rural and urban populations were affected and that the disease increased from what was a curiosity to becoming the most common cancer in black males in parts of South Africa within a mere 12 years.

The Zulus living in Natal, South Africa, showed similar increases during the late 1950s and early '60s (Schonland & Bradshaw, 1969). Hospital records from the early part of the century, as well as those derived from an early attempt at establishing a local cancer registry in 1905, did not reveal any cases of oesophageal cancer amongst Zulus. Confirmation of this low risk came from a study of Zulu migrants to the Johannesburg goldmines where no cases of oesophageal cancer occurred in Zulus prior to 1960 (Oettlé, 1967). Analysis of the incidence of oesophageal cancer during 1964–79 in goldminers from 10 widespread regions of southern Africa, however, showed that the rate was significantly raised above the mean for Zulus from Natal and for Xhosas from the well-known high incidence regions of Transkei and Ciskei (Bradshaw *et al.*, 1982). Since the increase was noted the incidence in Zulus has remained fairly stable apart from a possible slight increase over the last two decades. If the major factor in the aetiology is evident in the lifestyle then the cause of the disease may be expected to be a well-entrenched change from the traditional lifestyle.

This study was undertaken in an attempt to identify possible factors which might be associated with the causation of oesophageal cancer. It was designed to investigate many variables relating to lifestyle, such as those pertaining to socio-economic status, nutrition, exposure to suspected carcinogen sources, tobacco usage, alcohol consumption and traditional health medications.

### Materials and methods

Data were collected from 211 oesophageal cancer patients who were diagnosed at King Edward VIII Hospital, Durban, during the period 1978–1981. Interviews were conducted by a trained African social worker, using the vernacular of the patient. At the same time, data were collected from controls

*Present address: PO Box 13873, Sinoville 0129, South Africa.

Received 22 May 1984; and in revised form 22 November 1984.

selected from the hospital population to match each case by age and urban–rural background as these factors are known to be associated with oesophageal cancer. The controls were selected within a 5-year age group of each case, in such a way that the control could be up to 4 years older, but only one year younger than the case. Although the mean age of the cases was 54.3 years and that of the controls was 54.6 years, the difference was found to be significant on the basis of the matched pairs $t$-test ($t=2.40$; df$=210$; $P=0.0175$).

The rural nature of the area served by the hospital made a neighbourhood control study impossible. Use of hospital controls may, however, result in a higher frequency of factors that tend to promote hospitalization including factors which have been associated with the risk of oesophageal cancer such as poor nutrition, smoking and excessive alcohol use. Although this may therefore tend to make the study less sensitive for detecting these effects, the only alternative would have been to use various urbanized groups. In our experience, this would lead to an artificially elevated socio-economic status in the control group and its consequent distortions.

The success of a hospital control study depends on the referral patterns of the controls being the same as those of the cases. Although it was not possible to investigate this directly, the fact that the controls were selected from two different wards (surgical and medical) enabled a comparison of these two subgroups of controls. The group selected from the medical ward were found to differ significantly ($P<0.05$) on 9 out of the 273 variables. The differences indicated that the controls from the medical group were more rural than those from the surgical ward. A comparison of the cases matched to each of the two groups showed that these subgroups of cases also differed significantly on urban–rural factors. It was concluded that the observed differences could be attributed to the matching process and that at least the referral pattern of two subgroups of the controls was consistent.

The methods of analysis included McNemar's test for matched pairs on categorical variables, the matched pairs $t$-test, and the Wilcoxon matched pairs signed-ranked test on the continuous variables. A multivariate analysis was undertaken using stepwise logistic regression for matched data. The age difference was included as a factor at each step to take this difference into account. The FORTRAN programme, MATCH, written by Breslow & Day (1980) was used. This model was then used to estimate the relative risks (and 95% confidence limits) of different factors having adjusted for the effects of the other factors.

*Factors investigated*

Comparisons between the cases and controls were made on the factors mentioned below which were measured by 273 variables:

*Socio-economic factors* These factors included the school level attained by the individual and each parent, a weighted index of the level of education of the family, the marital status, number of children, number of siblings, number of rooms in house, the ownership of cattle, sheep, goats, pigs, chickens, ducks/geese/turkeys, dogs/cats, horses/mules/donkeys, ownership of agricultural land, ownership of house or kraal, the living conditions (whether backyard in township, backyard in white area, compound/hostel, township house, rural house of mud or brick), which type the individual lived in the longest and for how long, a weighted index over life-time, the number of people living in the same room, usage of fuel types (whether wood, manure, paraffin, spirits, coal/anthracite, gas or electricity), a weighted index, which was used for the longest and for what period, sleeping with an open-fire and if so the fuel type, the occupation (whether miner, farmer, unskilled labourer, semi-skilled labourer, artisan skilled labourer, clerical, managerial professional, self-employed or unemployed), the period of employment and a weighted index depending on the principal occupation, an index depending on the highest occupation attained and an index weighting each occupation according to length of service.

*Carcinogenic exposure* The period of exposure to the following possible carcinogens or potential carcinogens was investigated: petrol/tar/pitch/creosote/asphalt, silica dust, phytoliths, lead, asbestos, arsenic, soot, coal, insecticides and dynamite.

*Food* The frequency of eating particular foodstuffs was investigated. Included in the cereals were flour in white and brown bread, maize as purchased maize meal, homegrown maize meal, whole maize, bought samp (dehusked whole maize) or homegrown samp, grain sorghum or amarewa (soured drinks). Included in the fruit and vegetables were beans, imifino (wild spinach), cabbage, pumpkin/squash, peanuts, carrots, indumbe (yams), sweet potato, potato, tomato, onions, wild fruits and cultivated fruits. The animal proteins included red meat, chicken, fish, eggs, margarine, milk and amaas (soured milk). The beverages included tea with milk, tea without milk and coffee with or without milk.

*Health treatments* Treatment by traditional healers, medical doctors or both, was determined together with the use of herbal medicines as emetics, purgatives, external medication, internally for coughs or in any other way. The use of *umthuma* (*Solanum serdomeum*) for souring milk, as a medicine or for any other purpose, as well as the use of *kritsi* (*Argemone mexicana*), was investigated.

*Tobacco usage* The following habits were investigated: smoking pipes, commercial cigarettes, hand rolled cigarettes, marijuana cigarettes, chewing tobacco, *injonga* (pipe dottle), *isixaxa* (pipe ash) and snuff. In each case the type of tobacco used was investigated (i.e. whether home-grown, commercial, mixed or both), and if home-grown, the type as well as the method of curing (i.e. whether sun-dried, fire cured or other means). The total amount of tobacco used was investigated by taking into account the duration of smoking and the amount used per week. This was then weighted according to the age. The total amount of tobacco used in particular combinations was investigated viz. pipe with hand-rolled cigarettes, pipe with hand-rolled and commercial cigarettes, and *injonga* with *isixaxa*.

*Alcohol usage* The frequency of drinking, the duration of the habit, the amount per session, a subjective assessment of the habit and the total amount, taking duration, frequency, amount per session and age into account, was investigated for the following alcohols as well as particular combinations: *umgombothi* (home-brewed beer), *jabulani* (commercial traditional beer), Western beer/stout, wines, spirits, home-made spirits, concoctions, *umgombothi* with *jabulani,* Western beer/stout with wine and spirits, Western spirits with home-made spirits and home-made spirits with concoctions.

It should be noted that all the weighted indices were defined on an *a priori* basis.

### Results

All the men included in this study are Zulu (i.e. both parents were Zulu). The subjects were mainly born in Natal although 10 of them were born in neighbouring districts. Their ages ranged from 28 to 86 years. The age distributions of the cases and controls are presented in Table I.

When the cancer cases were compared with their controls they were found to differ significantly on 16 factors. These are shown in Table II in order of significance.

Logistic regression was then used to find out which of these factors could be used in combination to model the odds of being a cancer case rather than a control. Since the controls were significantly older than the cases, the age difference was included in the model. Pairwise interactions of these factors were also considered. The results of the logistic regression analysis are summarized in Table III which also shows the standardized regression coefficients for each factor included in the model. The results indicate that a model with four parameters in addition to the age parameter can be considered appropriate for the data as the addition of further parameters does not add significant information to the risk of being an oesophageal cancer case. None of the pairwise interactions were included in the model which suggests that the factors involved affect the risk independently.

The results show that smoking commercial cigarettes contributes most to the risk, then eating bought maize, smoking a pipe and eating margarine or butter less frequently. The relative risk (adjusted for effects of the other factors and the age difference) for each category of the factors in the model is presented in Table IV with the approximate 95% confidence limits.

### Discussion

Of likely relevance to the emergence of oesophageal cancer as a serious health problem among the Zulus are the socio-economic changes that have taken place over the past 50 years. Prior to this, the Zulu hunter-warrior lifestyle of antiquity had been replaced by a rural subsistence economy based on livestock and agriculture. Initially, indigenous African grains such as millet and sorghum were largely cultivated but these were gradually supplanted by high-yielding maize, a crop that, when supplemented with animal products, wild

**Table I** The age distribution of the 211 cancer cases and controls (as percentages)

| Age (y) | <45 | 45–49 | 50–54 | 55–59 | 60–64 | 65–69 | 70+ |
|---|---|---|---|---|---|---|---|
| Cases (%) | 17.1 | 17.5 | 16.1 | 16.1 | 16.1 | 9.0 | 8.1 |
| Controls (%) | 17.1 | 17.1 | 15.6 | 16.1 | 14.6 | 11.8 | 7.6 |

**Table II** The factors on which the cases and controls differed significantly (in order of significance)

| *Factor* | *Level* | *Case* (%) | *Control* (%) | *McNemar* | *P-value* |
|---|---|---|---|---|---|
| Commercial cigarettes | | | | | |
| | Yes | 72.5 | 51.7 | 22.4 | 0.0001 |
| | In past | 8.5 | 13.3 | | |
| | No | 19.0 | 35.1 | | |
| Amount of tobacco in commercial cigarettes (grams) | None | 21.8 | 36.5 | 21.43 | 0.0015 |
| | ≦20 | 18.5 | 18.5 | | |
| | 21–40 | 26.1 | 24.6 | | |
| | >40 | 33.6 | 20.4 | | |
| Bought maize | | | | | |
| | Daily | 96.2 | 87.7 | 12.53 | 0.0019 |
| | Weekly | 2.4 | 3.8 | | |
| | Less frequently | 1.4 | 8.5 | | |
| Amount of tobacco smoked in rolled cigarettes (grams) | None | 34.6 | 50.2 | 20.73 | 0.0021 |
| | ≦20 | 24.6 | 26.1 | | |
| | 21–40 | 22.3 | 14.7 | | |
| | >40 | 18.5 | 9.0 | | |
| Margarine or butter | | | | | |
| | Daily | 10.4 | 24.2 | 14.42 | 0.0024 |
| | Weekly | 44.1 | 35.5 | | |
| | Less frequently | 45.5 | 40.3 | | |
| Pipe smoking | | | | | |
| | Yes | 11.8 | 6.2 | 11.55 | 0.0091 |
| | In past | 14.7 | 7.6 | | |
| | No | 73.5 | 86.3 | | |
| Tea with milk | | | | | |
| | Daily | 75.8 | 67.3 | 11.07 | 0.0114 |
| | Weekly | 14.2 | 11.8 | | |
| | Less frequently | 10.0 | 20.9 | | |
| Rolled cigarettes | | | | | |
| | Yes | 53.6 | 41.2 | 10.61 | 0.0141 |
| | In past | 11.8 | 9.5 | | |
| | No | 34.6 | 49.3 | | |
| Rural mud house | | | | | |
| | Yes (owned or rented) | 41.7 | 30.8 | 5.69 | 0.0171 |
| | No | 58.3 | 69.2 | | |
| Amount of tobacco smoked in pipe (g) | | | | | |
| | None | 75.4 | 86.7 | 9.07 | 0.0283 |
| | ≦20 | 11.4 | 6.6 | | |
| | >20 | 13.3 | 6.6 | | |

**Table II** (continued)

| *Factor* | *Level* | *Case* (%) | *Control* (%) | *McNemar* | *P-value* |
|---|---|---|---|---|---|
| Family educational level index | | | | | |
| | None | 45.5 | 55.0 | 8.85 | 0.0314 |
| | ≦5 | 19.9 | 20.9 | | |
| | >5 | 33.6 | 23.2 | | |
| Total amount of homemade spirits and concoctions | None | 64.5 | 76.8 | 8.78 | 0.0323 |
| | ≦100 | 17.5 | 10.4 | | |
| | >100 | 18.0 | 12.8 | | |
| Ownership of agricultural land | | | | | |
| | Yes | 53.6 | 42.7 | 4.52 | 0.0335 |
| | No | 46.4 | 57.3 | | |
| Homemade spirits | | | | | |
| | Daily | 5.2 | 2.4 | 11.99 | 0.0348 |
| | Weekends | 4.7 | 7.1 | | |
| | Periodically | 10.4 | 5.7 | | |
| | Never | 74.9 | 80.1 | | |
| Own kraal | | | | | |
| | Yes | 35.5 | 26.5 | 4.25 | 0.0393 |
| | No | 64.5 | 73.5 | | |
| Schooling | | | | | |
| | Yes | 52.1 | 43.1 | 3.88 | 0.0488 |
| | No | 47.9 | 56.9 | | |

**Table III** Summary of the stepwise logistic regression and standardized regression coefficients for each parameter

| *Number of factors in model* | *Goodness of fit* | *Score-test* | *Age* | *Commercial cigarettes* | *Bought maize* | *Pipe* | *Margarine or butter* | *Own agricultural land* |
|---|---|---|---|---|---|---|---|---|
| 0 | 292.51 | | | | | | | |
| 1 | 286.57 | 5.61 | −2.29 | | | | | |
| 2 | 266.34 | 19.45 | −2.25 | 4.22 | | | | |
| 3 | 254.53 | 10.58 | −2.31 | 4.07 | 2.98 | | | |
| 4 | 249.36 | 5.09 | −2.41 | 3.85 | 2.84 | 2.21 | | |
| 5 | 244.47 | 4.81 | −2.24 | 3.67 | 2.77 | 2.31 | −2.17 | |
| 6 | 240.81 | 3.65 | −2.20 | 3.55 | 2.83 | 2.17 | −2.18 | 1.89 |

**Table IV** The relative risks based on the five parameter model having adjusted for the age difference

| *Factor* | *Level* | *Relative risk* | *Approximate 95% confidence limits* |
|---|---|---|---|
| Commercial cigarettes | Yes | 2.64 | (2.04; 3.42) |
| | In past | 1.62 | (1.25; 2.11) |
| | No | 1.00 | (0.77; 1.29) |
| Bought maize | Daily | 5.73 | (3.09; 10.63) |
| | Weekly | 2.39 | (1.29; 4.44) |
| | Less frequently | 1.00 | (0.54; 1.85) |
| Pipe smoking | Yes | 2.08 | (1.52; 2.84) |
| | In past | 1.44 | (1.06; 1.97) |
| | No | 1.00 | (1.73; 1.36) |
| Margarine or butter | Daily | 0.51 | (0.37; 0.69) |
| | Week-end | 0.71 | (0.52; 0.97) |
| | Less frequently | 1.00 | (0.74; 1.36) |

fruits and vegetables, probably constituted a well-balanced diet. More recently, population growth has outstripped the environment's capability to provide an adequate diet, and excessive maize dependence was manifested by large outbreaks of vitamin deficiency diseases, in particular pellagra (Warwick & Harington, 1973).

Economic pressures ultimately forced members of most households to seek external sources of income, largely by means of periodic migration to the cities for work. The resultant lifestyle, a combination of tribal links with industrial activity, represents a prolonged state of transitional westernization. A small proportion of the population has now become fully urbanised, and as a result now leads a typically Western way of life. This, however, was unusual 20–50 years ago when the initiating events of oesophageal cancer in the patients under study possibly occurred.

The results of this survey point to individuals in a transitional state as having the greatest susceptibility to the disease. Within the tribal context, those that develop the disease tended to be somewhat better educated, were more often the head of the extended tribal family and owned agricultural land. These advantages placed them in a better position to earn an income additional to the usual traditional subsistence economy and therefore made it more likely for them to purchase basic necessities. In this study, the cases used significantly more "purchased" maize meal, tea, cigarettes and tobacco. These items, together with sugar and salt (which were not studied here), represent the highest priority items for purchase over large parts of Africa. The more affluent members of the population buy more wheaten-bread, margarine or butter, vegetables, meat, soft drinks and coffee. The less affluent would have to rely on whole home-grown maize and a variety of items gathered from the environment.

In contrast to the adjacent Transkei, where pipe smoking was traditional, the Zulus did not produce tobacco for smoking. With urbanization they began smoking commercial cigarettes with avidity and now probably have the highest lung cancer rate in blacks of southern Africa (Bradshaw *et al.*, 1982). The increase of oesophageal cancer in Zulus by use of tobacco is indisputable although it must be appreciated that the mean total quantity of tobacco used is considerably less than that used by many affluent Caucasian populations where lung cancer is more common but oesophageal cancer is rare. The results of the present study agree with earlier investigations in Africa (Bradshaw & Schonland, 1974; Hunt, 1978) in showing that one in every five or six oesophageal cancer cases have never smoked.

Even more at variance with the usual pattern of risk factors found in the West is the apparent lack of appreciable effects of alcohol usage on the disease in Zulus, which is similar to observations made among Johannesburg blacks (Bradshaw & Schonland, 1974) and in Transkei (Rose, 1982). Rural blacks particularly usually use alcoholic beverages intermittently, at the most over weekends, and then only when grain for brewing is plentiful. Alcoholic cirrhosis is rare.

The results of this study appear to lend support to the existence of a nutritional predisposition to oesophageal cancer associated with a dietary staple low in vitamins and minerals (Van Rensburg, 1981). Relevant to this is the identification of a set of micronutrients that tends to be deficient in high-risk populations and that also significantly protects against experimentally-induced oesophageal carcinogenesis in rats (Van Rensburg, 1982; Van Rensburg *et al.*, 1983).

A high relative risk associated with the regular use of purchased maize meal found in this study is interesting since marked decreases in the concentration of four of the set of suspect nutrients (magnesium, zinc, nicotinic acid and riboflavin) in commercial maize meal, as opposed to whole maize, have been illustrated (Van Rensburg, 1981). In addition, every known high-risk population reviewed was shown to have subsisted for at least 50 years on either maize or wheat, both of which contain less minerals and vitamins than most other staple foods. Diets used by blacks in Washington DC that are characterised by a low vitamin and mineral density have also been found to have an extremely high relative risk for oesophageal cancer (Ziegler *et al.*, 1981).

The cause of the apparently reduced risk associated with the daily use of butter or margarine is unknown. Liberal use would contribute to the intake of fat soluble vitamins, particularly vitamin A. It is also possible that as the African diets are exceedingly low in oils, any additional fatty acids could enhance immunological responses as well as absorption of other nutrients, including minerals. Alternatively, it is possible that the association may be indirect, as for example, an index of affluence.

This study did not identify any other nutritious items that were used more frequently by the controls. It is possible that individual items may not have been detected due to the range of options available. Furthermore, it was not possible to defect quantitative differences with the methodology used in this study.

Rather revealing were the many factors exonerated in this study. These include various methods of traditional health care such as the use of herbal medicines, emetics, purgatives and the use of various wild plants. No association between the type of occupation, such as agricultural or industrial, or with any known source of possible carcinogens except for tobacco was established. The important factors associated with oesophageal cancer indicated in this study, – smoking and a diet low in micronutrients may reflect the difficulties and limitations of the transition to a western type of society. Rose (1979) has also noted a significantly elevated risk of oesophageal cancer in Transkeians who had become semi-westernized and whose circumstances are similar to those of the Zulu.

In conclusion, this case-control study has identified smoking and nutritional factors as being related to a high risk of oesophageal cancer. Although further studies are required to corroborate these findings, they suggest that a program discouraging smoking and improving nutrition should be undertaken to reduce the incidence of oesophageal cancer. Whereas these would be the eventual objectives they are difficult tasks and as an interim measure the vitamin and mineral enrichment of maize meal available to the population should be given consideration.

**References**

BRADSHAW, E., McGLASHAN, N.D., FITZGERALD, D. & HARINGTON, J.S. (1982). Analyses of cancer incidence in black gold miners from southern Africa (1964–79). *Br. J. Cancer*, **46**, 737.

BRADSHAW, E. & SCHONLAND, M. (1974). Smoking, drinking and oesophageal cancer in African males of Johannesburg, South Africa. *Br. J. Cancer*, **30**, 157.

BRESLOW, N.E. & DAY, N.E. (1980). *The Analysis of Case-Control Studies*. Lyon: International Agency for Research on Cancer, p. 298.

HUNT, J.A. (1978). Squamous cancer of the oesophagus in urban South African Blacks: A preliminary report of baseline studies. In: *Carcinoma of the Oesophagus*. (Ed. Silber), Cape Town: A.A. Balkema.

OETTLÉ, A.G. (1967). *Cancer Research in Africa*. Johannesburg: Witwatersrand University Press.

ROSE, E.F. (1979). Epidemiology of oesophageal cancer in southern Africa. *Adv. Med. Oncol. Res. Ed.*, **9**, 317.

ROSE, E.F. (1982). Esophageal cancer in Transkei – the pattern and associated risk factors. In: *Cancer of the Esophagus*. (Ed. Pfeifer), Florida: CRC Press, p. 19.

SCHONLAND, M. & BRADSHAW, E. (1969). Oesophageal cancer in Natal Bantu: A review of 516 cases. *S. Afr. Med. J.*, **43**, 1028.

VAN RENSBURG, S.J. (1981). Epidemiologic and dietary evidence for a specific nutritional predisposition to esophageal cancer. *J. Natl Cancer Inst.*, **617**, 243.

VAN RENSBURG, S.J. (1982). Oesophageal cancer, micronutrient malnutrition, and silica fragments. *Lancet*, ii, 1098.

VAN RENSBURG, S.J., BENADÉ, A.S., ROSE, E.F. & DU PLESSIS, J.P. (1983). Nutritional status of African populations predisposed to esophageal cancer. *Nutr. Cancer*, **4**, 206.

WARWICK, G.P. & HARINGTON, J.S. (1973). Some aspects of the epidemiology and etiology of esophageal cancer with particular emphasis on the Transkei, South Africa. *Adv. Cancer Res.* (Eds. Klein and Weinhouse), New York and London: Academic Press, p. 81.

ZIEGLER, R.G., MORRIS, L.E., BLOT, W.J., POTTERN, L.M., HOOVER, R. & FRAUMENT, J.F. Jr (1981). Esophageal cancer among black men in Washington, D.C. II. Role of nutrition. *J. Natl Cancer Inst.*, **67**, 1199.